# Conditionally Immortal *Slc4a11*^−/−^ Mouse Corneal Endothelial Cell Line Recapitulates Disrupted Glutaminolysis Seen in *Slc4a11^−/−^* Mouse Model

**DOI:** 10.1167/iovs.17-21781

**Published:** 2017-07

**Authors:** Wenlin Zhang, Diego G. Ogando, Edward T. Kim, Moon-Jung Choi, Hongde Li, Jason M. Tenessen, Joseph A. Bonanno

**Affiliations:** 1 School of Optometry, Indiana University, Bloomington, Indiana, United States; 2 Department of Biology, Indiana University, Bloomington, Indiana, United States

**Keywords:** Slc4a11 knockout, corneal endothelium, glutamine, ammonia

## Abstract

**Purpose:**

To establish conditionally immortal mouse corneal endothelial cell lines with genetically matched *Slc4a11*^+/+^ and *Slc4a11*^−/−^ mice as a model for investigating pathology and therapies for *SLC4A11* associated congenital hereditary endothelial dystrophy (CHED) and Fuchs' endothelial corneal dystrophy.

**Methods:**

We intercrossed *H-2Kb*-tsA58 mice (Immortomouse) expressing an IFN-γ dependent and temperature-sensitive mutant of the SV40 large T antigen (tsTAg) with *Slc4a11*^+/+^ and *Slc4a11*^−/−^ C57BL/6 mice. The growth characteristics of the cell lines was assessed by doubling time. Ion transport activities (Na^+^/H^+^ exchange, bicarbonate, lactate, and Slc4a11 ammonia transport) were analyzed by intracellular pH measurement. The metabolic status of the cell lines was assessed by analyzing TCA cycle intermediates via gas chromatography mass spectrometry (GC-MS).

**Results:**

The immortalized *Slc4a11*^+/+^ and *Slc4a11^−/−^* mouse corneal endothelial cells (MCECs) remained proliferative through passage 49 and maintained similar active ion transport activity. As expected, proliferation was temperature sensitive and IFN-γ dependent. *Slc4a11^−/−^* MCECs exhibited decreased proliferative capacity, reduced NH_3_:H^+^ transport, altered expression of glutaminolysis enzymes similar to the *Slc4a11^−/−^* mouse, and reduced proportion of TCA cycle intermediates derived from glutamine with compensatory increases in glucose flux compared with *Slc4a11*^+/+^ MCECs.

**Conclusions:**

This is the first report of the immortalization of MCECs. Ion transport of the immortalized endothelial cells remains active, except for NH_3_:H^+^ transporter activity in *Slc4a11*^−/−^ MCECs. Furthermore, *Slc4a11^−/−^* MCECs recapitulate the glutaminolysis defects observed in *Slc4a11^−/−^* mouse corneal endothelium, providing an excellent tool to study the pathogenesis of *SLC4A11* mutations associated with corneal endothelial dystrophies and to screen potential therapeutic agents.

*SLC4A11* mutations are associated with congenital hereditary endothelial corneal dystrophy (CHED), Fuchs' endothelial corneal dystrophy, Harboyan syndrome (CHED plus perceptive deafness), and Peters anomaly.^1–4^ Up to 80 distinct mutations in 17 of the 19 exons of *SLC4A11* have been identified in individuals with CHED,^3,5–17^ which is characterized at or soon after birth by bilateral diffuse corneal edema without other significant developmental abnormalities of the anterior segment.^18^ Histologically, the diffusely edematous corneal stroma accounts for the marked increase in corneal thickness observed clinically.^19^ In addition, CHED is associated with a uniform thickening of Descemet's membrane and vacuolization of corneal endothelium.^20^ The *Slc4a11*^−/−^ C57BL/6 mouse recapitulates the human CHED-related defects, exhibiting a similar ground-glass diffuse corneal edema, increased corneal thickness, vacuolated corneal endothelial cells, and uniformly thickened Descemet's membrane.^21,22^ Furthermore, similar to human CHED,^23^ endothelial cell density in *Slc4a11^−/−^* C57BL/6 mice is not significantly affected at early age.^22^ Based on these observations, both Han et al.^22^ and the authors of this study concluded that this *Slc4a11*^−/−^ C57BL/6 mouse can serve as a good animal model for human CHED.

Recent functional studies of SLC4A11 indicate that it is permeable to H^+24–26^ and can act as an NH_3_:H^+^ cotransporter.^24,25,27^ We recently demonstrated that this ammonia permeability is essential for human corneal endothelial cells, as these cells produce ammonia as a by-product of glutamine catabolism, which is required to maintain a high level of ATP production.^21^ Furthermore, we found that *Slc4a11*^−/−^ mouse corneal endothelium has disrupted expression of enzymes involved in glutamine metabolism.^21^ However, only a very limited amount of corneal endothelial material can be obtained from mice, constraining further cellular, molecular, and metabolic studies.

Of neural crest origin, differentiated corneal endothelial cells are arrested in the G1 phase of the cell cycle, and studies in both mice and humans have revealed that these cells have very low proliferative potential in vivo.^28^ When cultured in vitro, corneal endothelial cells manifest limited passaging ability with rapid senescence and epithelial-to-mesenchymal (EMT) transition.^29^ This limited proliferative potential makes obtaining sufficient corneal endothelial sample a significant challenge for studying the molecular mechanisms that underlie corneal physiology and the pathophysiology in corneal endothelial diseases.

To overcome these limitations, we intercrossed the *Slc4a11*^+/+^ and *Slc4a11*^−/−^ C57BL/6 mice^22^ with *H-2Kb*-tsA58 Immortomouse, which carries a temperature-sensitive mutant form of the simian virus-40 large T antigen.^30^ We used the resulting progeny to generate two conditionally immortal, genetically matched mouse corneal endothelial cell lines: *Slc4a11*^+/+^ mouse corneal endothelial cells (*Slc4a11*^+/+^ MCEC) and *Slc4a11*^−/−^ mouse corneal endothelial cells (*Slc4a11*^−/−^ MCEC). Here we report the successful establishment of these two genetically matched wild-type and *Slc4a11* knockout MCECs. We examined proliferative properties, transport activity, and Slc4a11-related glutaminolysis activity. The resultant cells retain key endothelial transport function and the Slc4a11-deficient cells show altered glutamine metabolism consistent with what was observed in *Slc4a11*^−/−^ mouse corneal endothelium.^21^ Overall, the generation of these cell lines establishes a valuable tool for studying aspects of corneal endothelial diseases that require a large number of cells.

## Methods

### Animal Genotyping

All mice were housed and maintained in specific pathogen-free conditions and used in the experiments in accordance with institutional guidelines and the current regulations of the National Institutes of Health, the US Department of Health and Human Services, the US Department of Agriculture, and the ARVO Statement for the Use of Animals in Ophthalmic and Vision Research.

The *H-2Kb*-tsA58 transgenic Immortomouse (Charles River Laboratories, Wilmington, MA, USA) was intercrossed with *Slc4a11*^+/+^ and *Slc4a11*^−/−^ C57BL/6 mice, respectively, to generate offspring bearing the Immortomouse transgene together with *Slc4a11*^+/+^ or *Slc4a11*^−/−^. *Slc4a11*^−/−^ C57BL/6 mice have a targeted deletion of exons 9 to 13 of the murine *Slc4a11* gene.^31^ The offspring were genotyped by PCR of genomic DNA from ear-punch sections following protocols using QIAamp DNA Mini Kit (Qiagen, Hilden, Germany). Oligomer sequences (5′–3′) used for PCR were as follows: *primer 1* (*Immorto*-F), AGT CCT CAC AGT CTG TTC ATG ATC; *primer 2* (null-F), GAT CTG CCT GAG GTG TTA CTT G; *primer 3* (null-R and *Immorto*-R, common reverse primer), GGA TGG CAT CAC TAG TCA TGA C; *primer 4* (*Slc4a11* wt-F), TCT GGA CTT CAA CGC CTT CT; *primer 5* (*Slc4a11* ko-F), GCC AAG GTA TGG AGA ACA CC; *primer 6* (*Slc4a11* wt-R and ko-R, common reverse primer), GCA CAA ACG TGA TGG AAA TG. Primers 1 and 3 were used to identify the SV40 transgene, *Immorto (tsTAg)*. Primers 2 and 3 were used to identify the null allele. *Primers 4* and *6* were used to identify the *Slc4a11* wild-type allele, and primers 5 and 6 were used to identify *Slc4a11* knockout allele. The first generations were bred, and the second-generation pups were genotyped by PCR to identify *Slc4a11^+/+^* and *Slc4a11^−/−^* animals that carried at least one copy of the SV40 *tsTAg* transgene to be used for tissue isolation.

### Generation of MCEC Cultures of *Slc4a11*^+/+^ and *Slc4a11*^−/−^ Genotypes

MCECs were prepared from 12-week-old mice. Briefly, the globes were aseptically enucleated followed by cornea dissection and corneal endothelium peeling. Then corneal endothelial sheets with attached Descemet's membrane were placed in OptiMEM-I medium (#51985; Thermo Fisher Scientific, Canoga Park, CA, USA) supplemented with 8% heat-inactivated fetal bovine serum (FBS) (#10082139; Thermo Fisher Scientific), EGF 5 ng/mL (#01-107, Millipore, Darmstadt, Germany), pituitary extract 100 μg/mL (Hyclone Laboratories, Logan, UT, USA), calcium chloride 200 mg/L, 0.08% chondroitin sulfate (#G6737; Sigma-Aldrich Corp., St. Louis, MO, USA), gentamicin 50 μg/mL (#15710072; Thermo Fisher Scientific), antibiotic/antimycotic solution diluted 1:100 (#15240062; Thermo Fisher Scientific) and 44 units/mL IFN-γ (#485-MI; R&D Systems, Minneapolis, MN, USA). IFN-γ was used to stimulate the MHC promoter for tsTAg expression. Cells were further selected based on morphology via single-cell cloning for colonies with corneal endothelial hexagonal shape and contact inhibition. Cells were incubated at 33°C with 5% carbon dioxide.

The two lines, *Slc4a11*^+/+^ MCEC and *Slc4a11*^−/−^ MCEC, were genotyped by PCR following protocols using QIAamp DNA Mini Kit (Qiagen). Primer sequences used were the same as the primers used for mouse genotyping listed above.

### Cell Expansion and Continuous Propagation

MCECs were cultured at 33°C (5% CO_2_) or 37°C (5% CO_2_) in OptiMEM-I medium (51985; Thermo Fisher Scientific) supplemented with 8% heat-inactivated FBS (10082139; Thermo Fisher Scientific), EGF 5 ng/mL (01-107; Millipore), pituitary extract 100 μg/mL (Hyclone Laboratories), calcium chloride 200 mg/L, 0.08% chondroitin sulfate (G6737, Sigma-Aldrich Corp.), gentamicin 50 μg/mL (15710072; Thermo Fisher Scientific), antibiotic/antimycotic solution diluted 1:100 (15240062; Thermo Fisher Scientific) and with or without 44 units/mL IFN-γ (485-MI; R&D Systems). Culture at 33°C with IFN-γ is defined as permissive growth condition, whereas culture at 37°C without IFN-γ is defined as nonpermissive growth condition.

### Light Microscopy

Cell morphology images were acquired with an Infinity I camera (Lumenera Corp., Ottawa, ON, Canada) attached to an inverted phase-contrast microscope.

### Growth Curve and Doubling Time

MCEC cells were seeded at 5 × 10^3^ or 1 × 10^4^/mL (total 2500 cells/well or 5000 cells/well) in 24-well plates. Cells in four wells of 24-well plates were trypsinized to suspension and counted with a Cellometer Auto T4 (Nexcelom Bioscience, Lawrence, MA, USA). Doubling time was calculated using GraphPad Prism 6.1c (GraphPad Software, Inc., La Jolla, CA, USA).

### Western Blot of SV40 Large T Antigen

MCECs were seeded at 5 × 10^4^/mL (total 1 × 10^5^ cells/well) in six 35-mm dishes and cultured with IFN-γ at 33°C to subconfluence. While one dish was subject to protein extraction as the sample for the permissive condition, the remaining five dishes were switched to 37°C without IFN-γ for the subsequent nonpermissive culture. In the following 5 days, one dish was subjected to protein extraction on each day (24, 48, 72, 96, and 120 hours) in the nonpermissive condition. Cells in each dish were washed two times with ice-cold PBS and suspended in 100 μL RIPA buffer (50 mM Tris base, 150 mM NaCl, 0.5% deoxycholic acid-sodium salt, 2% SDS, and 1% NP40, pH 7.5) with Complete Protease Inhibitor Cocktail (#4693159001; Roche Diagnostics, Indianapolis, IN, USA). Then the sample was sonicated and centrifuged for 20 minutes at 12000*g* at 4°C. Cell lysate samples (15 μL) were mixed with Protein Loading Buffer Blue (2X) (EC-886, National Diagnostics, Atlanta, GA, USA) and a total volume of 30 μL was resolved on 1.5-mm-thick 10% SDS-polyacrylamide gels and wet-transferred to polyvinylidene difluoride membranes (Bio-Rad, Hercules, CA, USA). Membranes were blocked with 5% nonfat milk in TBST (25 mM Tris base, 137 mM NaCl, 0.1% Tween20) and probed with primary antibodies in the same buffer overnight at 4°C. The following primary antibodies were used: rabbit anti-SV40 T Ag 1:5000 (sc-20800; Santa Cruz Biotechnology, Dallas, TX, USA); and anti–glyceraldehyde 3-phosphate dehydrogenase antibody 1:1000 (sc-32233; Santa Cruz Biotechnology). Next, membranes were probed with secondary antibody (goat anti-rabbit IgG peroxidase-conjugated antibody, #A0545, or goat anti-mouse IgG peroxidase-conjugated antibody, #A8924; Sigma-Aldrich Corp.) for an hour at room temperature. Bound secondary antibodies were detected using an enhanced chemiluminescence assay (Supersignal West Pico, #34080; Thermo Fisher Scientific). Band densities with background subtraction were quantified using ImageJ (http://imagej.nih.gov/ij/; provided in the public domain by the National Institutes of Health, Bethesda, MD, USA).

### Intracellular pH (pH_i_) Measurement

pH_i_ measurements were performed as described previously.^32^ Briefly, MCECs were cultured on poly-L-lysine and fibronectin precoated 25-mm diameter glass coverslips (GG-25-pdl; Neuvitro Corporation, Vancouver, WA, USA) for 2 to 3 days in semipermissive condition. Before each experiment, cells were incubated with 10 μM of pH-sensitive fluorescent dye BCECF-AM (2′7′-bis(carboxyethyl)-5(6)-carboxyfluorescein-acetoxymethyl ester, B1170; Thermo Fisher Scientific) in Ringer's solution for 30 minutes at room temperature, and washed in dye-free Ringer's solution for another 30 minutes. The Ringer's solution constitution is listed in Table 1. All of the experimental solutions were equilibrated with air (or 5% CO_2_ for bicarbonate-rich [BR] solutions) and adjusted to pH 7.5 with 1N NaOH at 37°C. Osmolarity of all solutions was adjusted to 295 to 300 mOsm with mannitol. Coverslips with subconfluent cells were mounted into a perfusion chamber, and the chamber was then placed on a stage warmer (37°C) of an inverted microscope (Eclipse TE200; Nikon, Tokyo, Japan). Solutions were kept at 37°C in a warming box, and the flow of the perfusate (∼0.5 mL/min) was achieved by gravity. Cells were imaged with an oil-immersion objective (×40; Nikon). BCECF fluorescence was excited alternately at 495 ± 10 nm and 440 ± 10 nm, and the emitted light was collected through a bandpass filter (520–550 nm). Fluorescence ratios (495/440) were obtained at 1 Hz, and converted into pH_i_ using the high K^+^/nigericin calibration approach.^33^

**Table 1:**
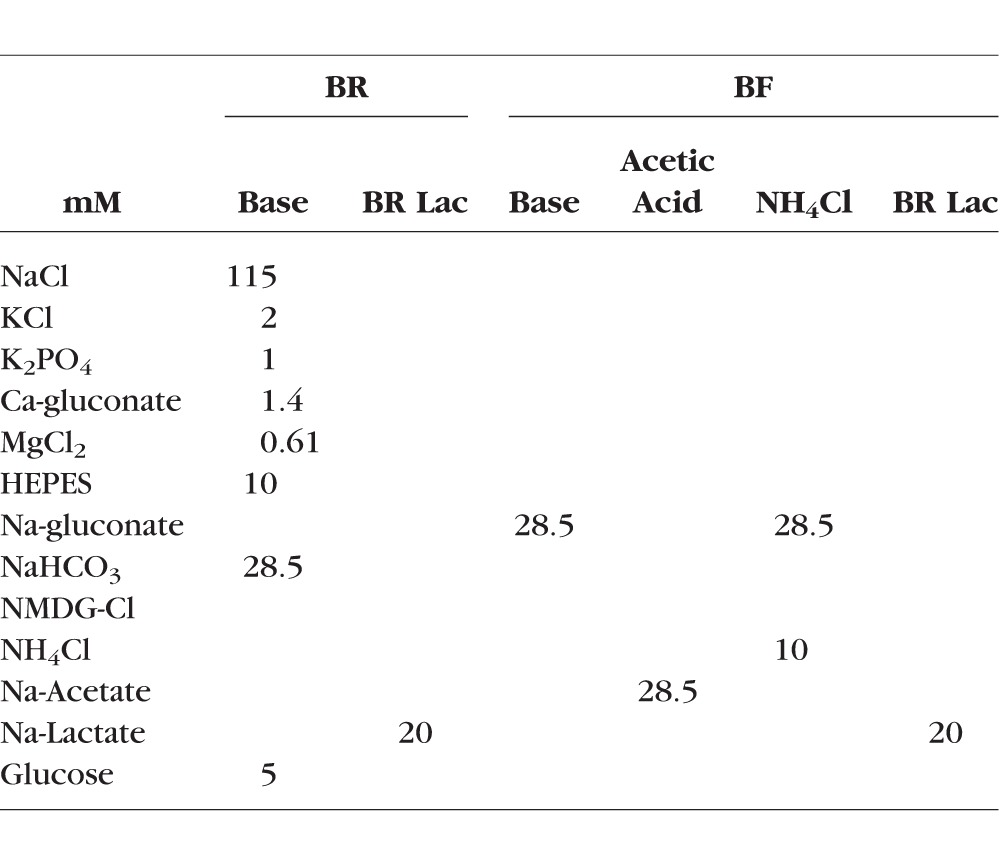
Composition of Extracellular Solutions, mM

### RNA Extraction, RT-PCR, and Real-Time Quantitative PCR

Total RNA from the MCEC cell lines (cultured in 33°C, IFN-γ [+]) was extracted and purified using the RNeasy mini kit (#74104; Qiagen) with DNase digestion (#79254; Qiagen). Complementary DNA was generated with a High Capacity RNA-to-cDNA Kit (Applied Biosystems, Foster City, CA, USA) at 10 ng RNA/μL reverse transcription reaction concentration. Real-time quantitative PCR reactions were set up in triplicate using PowerUp SYBR Green Master Mix (A25741; Thermo Fisher Scientific). Reactions were performed with murine gene primers listed in Table 2. A 2^−ΔΔCt^ experimental design was used for relative quantification and normalized to mouse ACTB (β-actin) for differential expression levels of target genes.

**Table 2:**
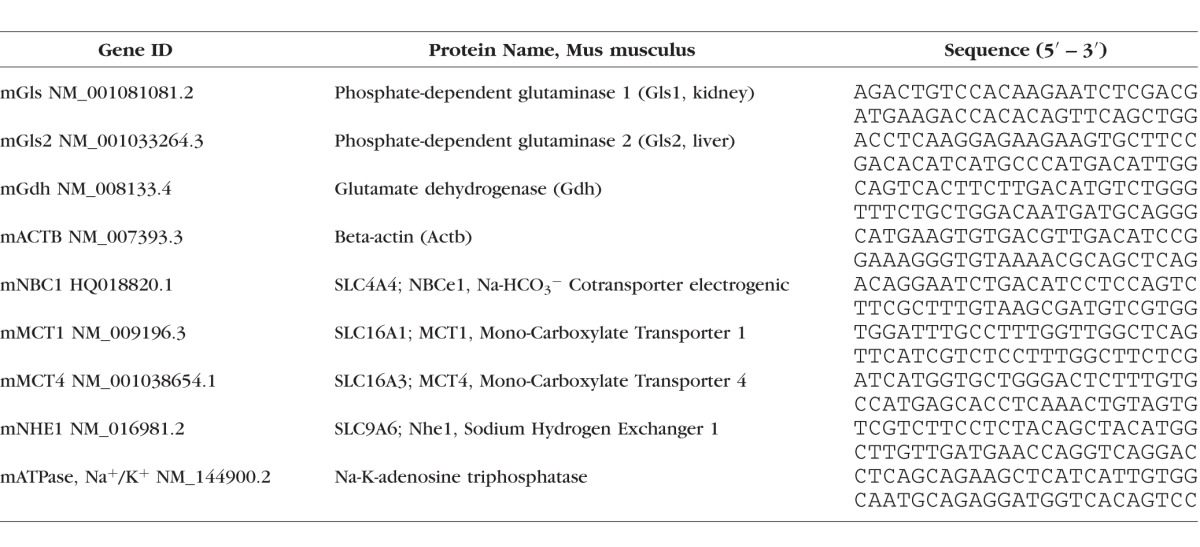
Murine Gene Primers

### Metabolites Extraction and Gas Chromatography Mass Spectrometry (GC-MS) Measurements

*Slc4a11*^+/+^ and *Slc4a11*^−/−^ MCEC in 8% FBS OptiMEM-I in T-75 flasks seeded at 5 × 10^4^/mL (total 2.5 × 10^5^ cells) were grown to confluence for 4 days at 33°C with IFN-γ then switched to serum-free OptiMEM-I supplemented with 200 mg/L CaCl_2_ and 0.08% chondroitin sulfate for 12 hours. Then the cells were incubated with serum-free Dulbecco's modified Eagle's medium (DMEM) conditional medium with 2.5 g/L U-^13^C_6_-D-glucose (CLM-1396-0; Cambridge Isotope Laboratories, Tewksbury, MA, USA) + 4 mM L-glutamine and incubated for 12 hours. Serum-free DMEM conditional medium was made using DMEM (glucose-free, glutamine-free, pyruvate-free, GIBCO #A1443001; Thermo Fisher Scientific) supplemented with CaCl_2_ 200 mg/L, 0.08% chondroitin sulfate (Sigma-Aldrich Corp.), and the isotope-labeled glucose and nonlabeled glutamine (Sigma-Aldrich Corp.). After 12-hour incubation, isotope-labeled cells were washed three times with ice-cold 0.9% NaCl, quenched with 2 mL ice-cold 100% methanol, scraped off the plate and removed together with 2 mL quenching methanol into centrifuge tubes, vortexed thoroughly, and centrifuged at 8000*g* for 2 minutes. Supernatants were collected and the cell pellet was resuspended with 900 μL 90% methanol twice following vortex and centrifugation. Total supernatant was kept stationary at −20°C for 1 hour, then centrifuged at 15,000*g* for 5 minutes at 4°C. The supernatant was collected to new tubes and dried in a centrifugal evaporator at room temperature overnight. The level of each metabolite isotopologue was measured using GC-MS as reported previously.^34^ The fraction of each isotopologue and the contribution of ^13^C to the total carbon pool of each metabolite was calculated as previously reported.^35^ Briefly, the isotopologue distributions were corrected based on the natural abundance of elements, and the fraction is the contribution of each isotopologue to the total abundance of all the isotopologues. The contribution of ^13^C to the total carbon pool of each metabolite is the weighted average of all the labeled isotopologues according to the fraction distribution.

### Statistical Analysis

Statistical analysis was carried out using GraphPad Prime 6.0c (GraphPad Software, Inc., La Jolla, CA, USA). Student's *t*-test or paired *t*-test were used for two-group comparison. Two-way ANOVA was used for metabolite data.

## Results

### Conditional Immortal *Slc4a11*^+/+^ MCEC and *Slc4a11*^−/−^ MCEC Growth Characteristics

**Figure 1:**
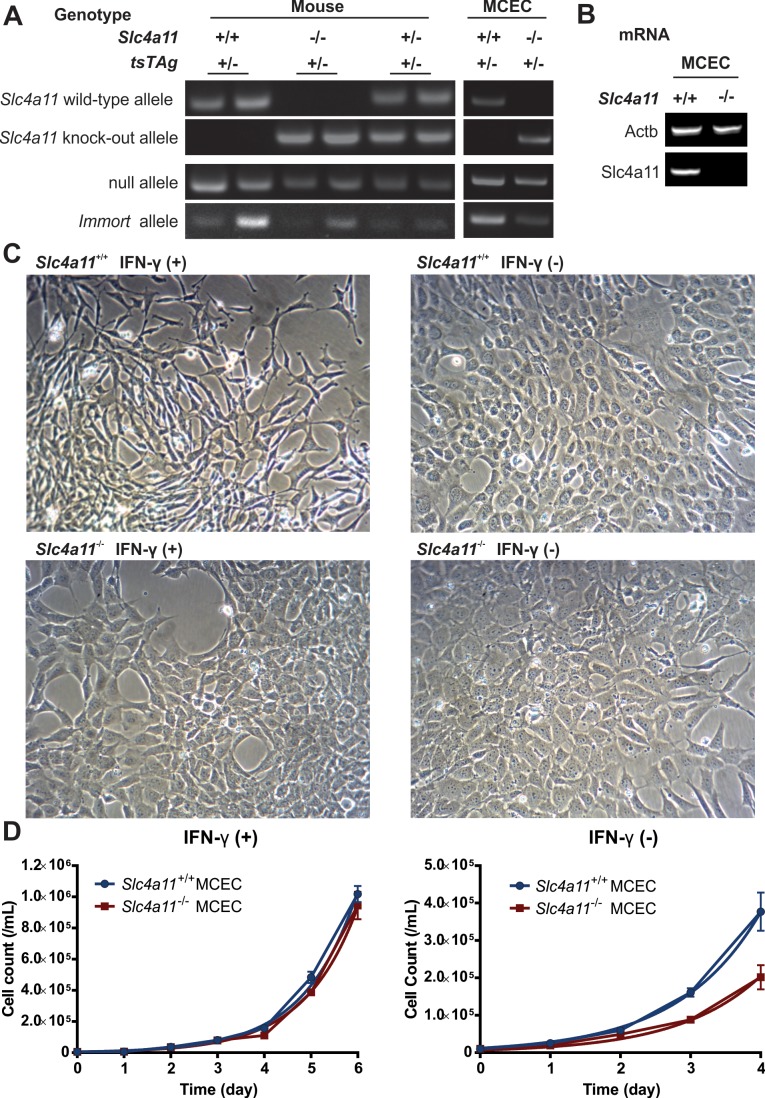
Characterization of *Slc4a11*^+/+^ and *Slc4a11*^−/−^ MCECs. (**A**) Genotypes of mice used for corneal endothelial cell collection, and genotypes of cultured MCECs. *Slc4a11* wild-type (wt) and knockout (ko) alleles were identified with specific primers yielding products of 353 bp and 386 bp, respectively. *Immorto (tsTAg)* and null alleles yielded PCR products of 500 bp and 300 bp, respectively. *Slc4a11*^+/+^ and *Slc4a11*^−/−^ mice with one allele of *Immorto* gene were used for cell derivation. *Slc4a11*^+/+^ and *Slc4a11*^−/−^ MCECs show the expected genotype of *Slc4a11* and *tsTAg* gene. (**B**) RT-PCR of *Slc4a11*^+/+^ and *Slc4a11*^−/−^ MCECs verified that no *Slc4a11* mRNA is expressed in ko. (**C**) Morphology of *Slc4a11*^+/+^ and *Slc4a11*^−/−^ MCECs cultured at 33°C with and without IFN-γ. (**D**) Growth curve of *Slc4a11*^+/+^ and *Slc4a11*^−/−^ MCECs cultured at 33°C with and without IFN-γ.

The genotypes of both the mice used for cell expansion (Fig. 1A, left panel) and of the derived MCECs (Fig. 1A, right panel), *Slc4a11*^+/+^ MCECs, and *Slc4a11*^−/−^ MCECs, were determined by PCR analysis of mouse or cell line genomic DNA. For the mice used, *Slc4a11* wild-type alleles are present in *Slc4a11*^+/+^ mice (left two lanes) and in *Slc4a11*^+/−^ mice (right two lanes), whereas *Slc4a11* knockout alleles are present in *Slc4a11*^−/−^ mice (middle two lanes) and in *Slc4a11*^+/−^ mice. For *Immorto* (*tsTAg*) gene, all of the mice we used carry only one allele of the gene, and the other allele is detected as a null allele. We selected *Slc4a11*^+/+^ *Immorto*^+/−^ mice and *Slc4a11*^−/−^ *Immorto*^+/−^ mice for cell expansion. Genotypes of derived cell lines were confirmed by PCR of the cell line genomic DNA for *Slc4a11* and *Immorto (tsTAg)* genes (Fig. 1A, right panel). Verification of *Slc4a11* mRNA expression in the cell lines was carried out by RT-PCR showing only *Slc4a11*^+/+^ but not *Slc4a11*^−/−^ MCEC expresses *Slc4a11* mRNA (Fig. 1B).

Next, we examined the cell morphology and growth doubling time of *Slc4a11*^+/+^ MCECs and *Slc4a11*^−/−^ MCECs. The two cell lines remained proliferative to passage 49 and are still propagating. We noticed a fibroblastic-like cell morphology in *Slc4a11*^+/+^ MCECs cultured under permissive conditions (IFN-γ [+], 33°C), in which the cells are elongated and lacking contact inhibition at high density. However, *Slc4a11*^−/−^ MCECs had a more hexagonal shape closely resembling corneal endothelial primary culture and form a monolayer when confluent in the same IFN-γ (+) medium (Fig. 1C). We asked if IFN-γ stimulated SV40 large T antigen (tsTAg) expression changes the MCEC morphology, so IFN-γ was removed, but the cells were kept at 33°C to see if the morphology was affected in *Slc4a11*^+/+^ MCECs. We define this 33°C IFN-γ (−) culture condition as “semipermissive.” Indeed, in the semipermissive condition, *Slc4a11*^+/+^ MCECs presented with a more hexagonal shape and cell monolayer was formed once confluent (Fig. 1C). In contrast, *Slc4a11*^−/−^ MCEC morphology in semipermissive culture was similar to that in permissive culture (Fig. 1C). In terms of growth, although there is a small difference (*P* = 0.013) in the doubling time between *Slc4a11*^+/+^ MCECs (0.78 day) and *Slc4a11*^−/−^ MCECs (0.79 day) in the permissive condition, *Slc4a11*^−/−^ MCECs (doubling time: 0.93 day) grow significantly slower than *Slc4a11*^+/+^ MCECs (0.76 day) in the semipermissive condition (*P* < 0.0001) (Fig. 1D).

**Figure 2:**
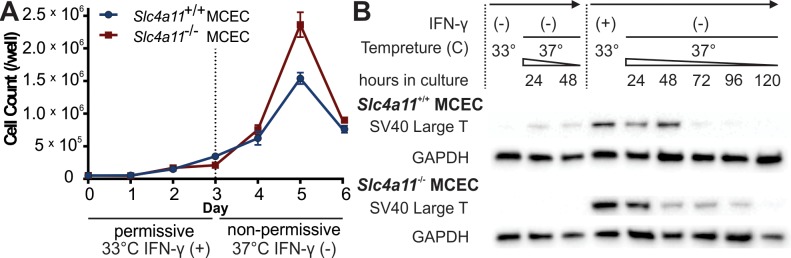
SV40 expression and growth characteristics in permissive, semipermissive, and nonpermissive conditions. (**A**) Growth profile of *Slc4a11*^+/+^ MCECs and *Slc4a11*^−/−^ MCECs when transferred from permissive culture (33°, IFN-γ [+]) to nonpermissive culture (37°C, IFN-γ [−]). (**B**) Western blot analysis of SV40 large T antigen expression in *Slc4a11*^+/+^ MCECs and *Slc4a11*^−/−^ MCECs under semipermissive culture (33°, IFN-γ [−], 4 weeks), and from permissive culture (33°, IFN-γ [+]) to nonpermissive culture (37°, IFN-γ [−]), showing a decrease of SV40 in nonpermissive culture over time.

Removal of IFN-γ reduces stimulation of the HMC promoter constructed upstream of the *Immorto (tsTAg)* gene. Given the dynamic SV40 large T antigen expression changes expected from IFN-γ withdrawal and change in culture temperature, we next analyzed the growth behavior of the MCECs under permissive (33°C, IFN-γ [+]) and subsequent nonpermissive conditions (37°C, IFN-γ [−]). In nonpermissive culture, in addition to the lack of IFN-γ stimulation on the HMC promoter, a shift to the 37°C incubation temperature induces degradation of the tsTAg mutant protein. As a result, MCEC proliferation gradually stopped once the culture was removed from the permissive condition into the nonpermissive condition (Fig. 2A). In addition, we also performed Western blot to analyze the SV40 large T antigen level in this series in comparison to SV40 large T level in semipermissive culture (33°C, IFN-γ [−]) (Fig. 2B). Both *Slc4a11*^+/+^ and *Slc4a11*^−/−^ MCECs showed very little SV40 large T antigen at 4 weeks of semipermissive culture and temperature maneuver minimally affected the SV40 large T level (Fig. 2B, first three lanes). The SV40 large T antigen level was highest in the permissive culture (33°C, IFN-γ [+]). When immediately moved to the nonpermissive culture (37°C, without IFN-γ), there was a time-dependent decline in large T antigen level, in parallel with the decline of cell proliferation (Fig. 2A, 2B). To summarize, semipermissive culture of *Slc4a11*^+/+^ MCECs and *Slc4a11*^−/−^ MCECs maintains cell proliferation and corneal endothelial morphology with declining level of SV40 large T antigen expression. Due to the rapid decline of cell proliferation and accompanied large cell loss within the first 2 days of moving from 33°C permissive to 37°C nonpermissive culture, which could introduce large variances in experimental results, together with good endothelial morphology and declining large T antigen levels in the semipermissive condition (33°C, IFN-γ [−]), we chose to test the ion transporter properties of these cells in the semipermissive state.

#### *Slc4a11*^+/+^ and *Slc4a11*^−/−^ MCEC Have Similar Active Ion Transport Activity

**Figure 3:**
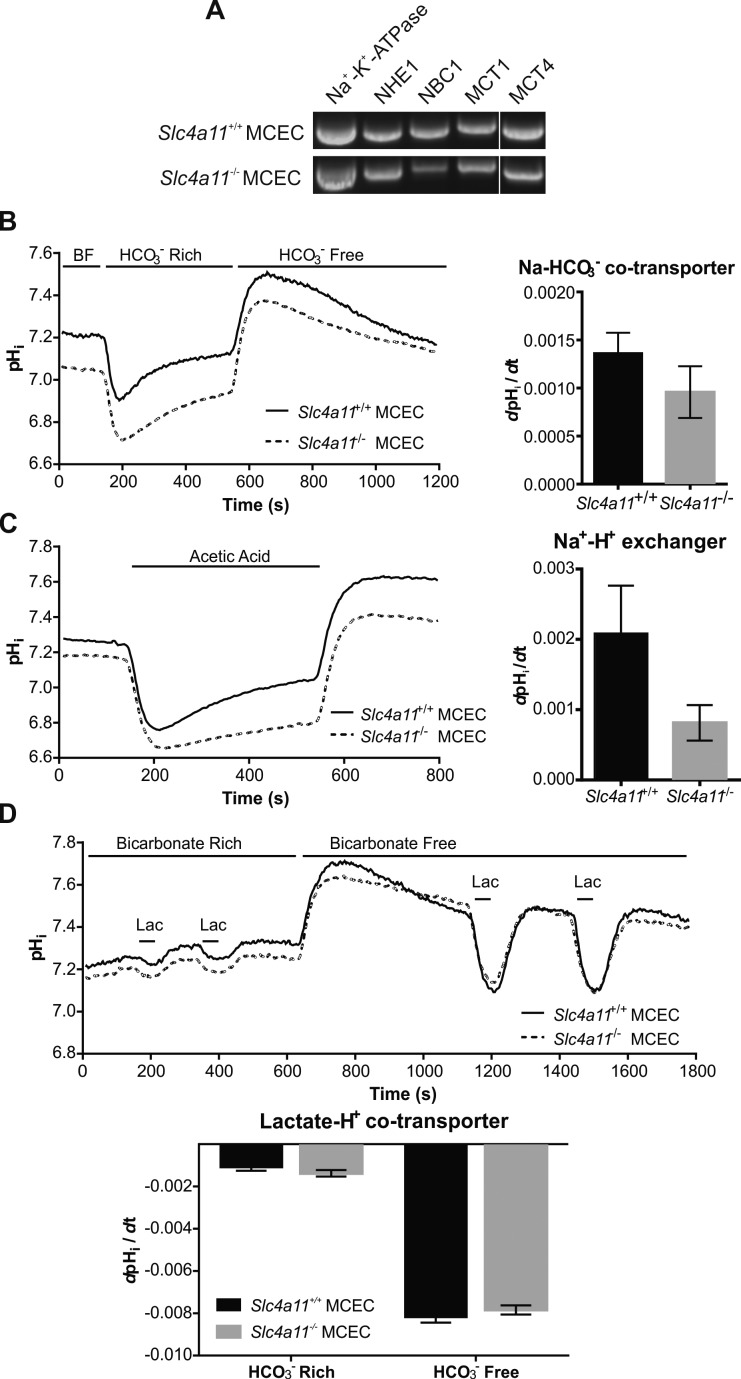
Na^+^/H^+^ exchanger, Na^+^-HCO_3_^−^ cotransporter, and H^+^-lactate cotransporter activity in *Slc4a11*^+/+^ and *Slc4a11*^−/−^ MCECs. (**A**) RT-PCR analysis of selected transporter mRNA expression in *Slc4a11*^+/+^ MCECs and *Slc4a11*^−/−^ MCECs, including Na^+^-K^+^-ATPase, Na^+^/H^+^ exchanger (NHE1), Na^+^-HCO_3_^−^ cotransporter (NBC1), and H^+^-lactate cotransporter (MCT1 and MCT4). (**B**) Analysis of apparent bicarbonate transporter activity. (**C**) Analysis of apparent Na^+^/H^+^ exchanger activity. (**D**) Analysis of apparent lactate transporter activity. *Bar graphs* show summary statistics.

To verify that these MCECs still carry significant ion transport activities, we next examined the mRNA expression and transport activity of selected ion transporters. RT-PCR analysis of *Slc4a11*^+/+^ and *Slc4a11*^−/−^ MCECs revealed that these cells express mRNA encoding the essential transporters Na^+^-K^+^-ATPase, Na^+^/H^+^ exchanger (SLC9A6; NHE1, Sodium Hydrogen Exchanger 1), Na^+^-HCO_3_^−^ cotransporter (SLC4A4; NBCe1, Sodium Bicarbonate Cotransporter electrogenic), H^+^-Lactate^−^ cotransporters (SLC16A1; MCT1, Mono-Carboxylate Transporter 1; and SLC16A3; MCT4, Mono-Carboxylate Transporter 4) (Fig. 3A). Similarly, BCECF-based continuous pH_i_ monitoring of apparent proton fluxes indicate the activity of bicarbonate transporters (Fig. 3B), Na^+^/H^+^ exchangers (Fig. 3C), and lactate transporters (Fig. 3D) are present and comparable between the *Slc4a11*^+/+^ and *Slc4a11*^−/−^ MCECs (see below).

Sodium Bicarbonate Cotransporter: In Figure 3B left panel, cells were initially perfused with bicarbonate-free (BF) Ringer's and then changed to BR ([HCO_3_^−^] 28.5 mM) Ringer's. Due to simple diffusion of dissolved CO_2_ inward across the plasma membrane, the cell rapidly acidifies as CO_2_ reacts with intracellular H_2_O to form H_2_CO_3_ and further release one H^+^ as a weak acid. Then pH_i_ slowly rises, which indicates the Na-HCO_3_^−^ cotransporters are moving weak base HCO_3_^−^ inward using Na^+^ inward transmembrane electrochemical gradient. We determined the initial slope of this pH_i_ rise as an indirect measure of the apparent Na^+^-HCO_3_^−^ cotransport activity. This assay revealed no significant difference between *Slc4a11*^+/+^ and *Slc4a11*^−/−^ MCECs in apparent bicarbonate transporter activity (Fig. 3B, Student's *t*-test, *P* = 0.48, *n* = 3 in each group). The overshoot after CO_2_/HCO_3_^−^ removal is an additional indication that there was HCO_3_^−^ accumulation inside the cell during BR perfusion.

Na^+^/H^+^ exchanger: In Figure 3C left panel, cells were initially perfused with BF Ringer's and then switched to BF Ringer's containing 28.5 mM Na-Acetate. Once dissolved in solution, a small fraction of Na-Acetate becomes noncharged acetic acid that readily diffuses across the membrane and rapidly acidifies the cell cytosol. Then pH_i_ recovers due to the activity of Na^+^/H^+^ exchangers and Slc4a11 proton permeability.^25^ Here, we measured the initial slope of this pH_i_ recovery as an indirect measure of the apparent Na^+^/H^+^ exchanger activity, revealing that there is a borderline significant difference between *Slc4a11*^+/+^ and *Slc4a11*^−/−^ MCECs (Fig. 3C, paired *t*-test, 2-tailed *P* = 0.10, 1-tailed *P* = 0.05, *n* = 3 in each group) that can be attributed to Slc4a11 proton permeability.^25^

H^+^-lactate^−^ cotransporter: Because we recently demonstrated that lactate transport is a significant component of the corneal endothelial pump,^36^ we next examined the lactate-dependent H^+^ fluxes in MCECs. Cells were initially perfused with BR Ringer's, and where indicated (horizontal bars), 20 mM lactate (dissolved in BR Ringer's, pH 7.4) was applied twice for 60 seconds (Fig. 3D, upper panel). The cells were then switched to perfusion with BF Ringer's and again 20 mM lactate (dissolved in BF Ringer's, pH 7.4) was applied twice for 60 seconds. Lactate exposure (pH 7.4) induces intracellular acidification due to the activity of H^+^-lactate cotransporters (Mono-Carboxylate Transporters, MCTs),^37^ in which the lactate transmembrane inward gradient favors the inward movement of lactate and H^+^ together causing intracellular H^+^ accumulation. The acidification is less prominent in BR than in BF Ringer's because HCO_3_^−^/CO_3_^2−^ offers extra H^+^ buffering power. We measured the initial slope of the induced acidification on lactate perfusion as an indirect measure of the apparent H^+^-lactate cotransporter activity. Figure 3D lower panel shows there was no significant difference between *Slc4a11*^+/+^ and *Slc4a11*^−/−^ MCECs in both BF and BR Ringer's (*t*-test, in BR *P* = 0.25, in BF *P* = 0.35, *n* = 3 in each group).

#### Slc4a11 NH_3_:H^+^ Flux Is Intact in *Slc4a11*^+/+^ MCECs But Impaired in *Slc4a11*^−/−^ MCECs

**Figure 4:**
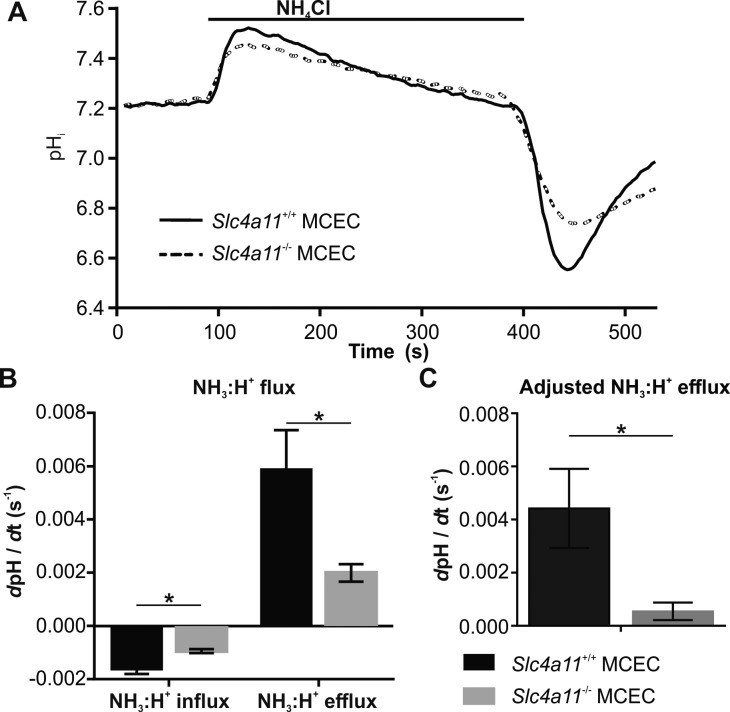
NH_3_:H^+^ Flux in *Slc4a11*^+/+^ and *Slc4a11*^−/−^ MCECs. (**A**) Analysis of NH_3_:H^+^ cotransporter activity in *Slc4a11*^+/+^ MCECs and *Slc4a11*^−/−^ MCECs. Cells loaded with the pH-sensitive dye BCECF were perfused in BF Ringer's and 10 mM NH_4_Cl was applied for 5 minutes. (**B**) Apparent NH_3_:H^+^ influx (*P* = 0.009, gradual acidification phase during NH_4_Cl pulse) and NH_3_:H^+^ efflux (*P* = 0.035, intracellular pH recovery phase after NH_4_Cl pulse) were both impaired in *Slc4a11*^−/−^ MCECs compared with *Slc4a11*^+/+^ MCECs. (**C**) Adjusted NH_3_:H^+^ efflux was calculated from apparent NH_3_:H^+^ efflux subtracted by apparent Na^+^/H^+^ exchanger-mediated H^+^ efflux. Adjusted NH_3_:H^+^ efflux is close to zero in *Slc4a11*^−/−^ MCECs. *P < 0.05.

We recently reported that human SLC4A11 mediates electrogenic transmembrane NH_3_:H^+^ fluxes,^27^ and this result has been verified by two other independent groups.^25,26^ Furthermore, a recent report from a third group shows murine Slc4a11 mediates similar electrogenic transmembrane NH_3_:H^+^ fluxes.^24^ So we next set out to determine if the Slc4a11-mediated NH_3_:H^+^ flux is impaired in *Slc4a11*^−/−^ MCECs (Fig. 4A). Cells were initially incubated with BF Ringer's, pulsed with 10 mM NH_4_Cl in BF Ringer's, and then switched back to BF Ringer's. Once dissolved in solution, a small fraction of NH_4_Cl forms NH_3_, a small noncharged molecule that is readily membrane diffusible. On entry, NH_3_ instantaneously reacts with intracellular H_2_O to form NH_4_^+^ and releases OH^−^. The latter rapidly alkalinizes the cell, causing the initial rapid alkalinizing phase (100–120 seconds) on NH_4_Cl application. Eventually the cell will reach an equilibrium where intracellular [NH_3_] equals extracellular [NH_3_]. Then a slow acidification occurs in the midphase (120–400 seconds) of the NH_4_Cl pulse, indicating there is an additional weak acid NH_4_^+^ (or NH_3_:H^+^ equivalently) flux entering the cell.^38^ In this case, Slc4a11 activity brings more NH_3_:H^+^ into the cell. Figure 4B shows that the rate of this slow acidification is significantly faster in *Slc4a11*^+/+^ MCECs compared with *Slc4a11*^−/−^ MCECs, consistent with the additional NH_3_:H^+^ influx provided by Slc4a11. Then, when the NH_4_Cl was washed away by BF Ringer's, there is a pronounced and rapid acidification on NH_4_Cl removal (400–440 seconds) (Fig. 4B). This is due to the rapid exit of NH_3_ gas and the conversion of accumulated NH_4_^+^ to NH_3_ + H^+^. The rapid acid loading immediately after NH_4_Cl removal is a reflection of the amount of weak acid (NH_4_^+^ or NH_3_:H^+^) that has entered the cell during the NH_4_Cl pulse.^38^ And even though NH_4_Cl was removed in this phase on the outside, there is NH_4_^+^ temporarily trapped intracellularly.^38^ The amount of NH_4_^+^ trapped is directly correlated with the extent of the acidification according to the Henderson-Hasselbalch equation.^38^ Figure 4A shows that the depth of this acid load is much greater in *Slc4a11*^+/+^ MCECs. The pH_i_ recovery (440–520 seconds) from this acid load is a phenomenon of the collective effect from Slc4a11-mediated NH_3_:H^+^ efflux and Na^+^/H^+^ exchanger-mediated H^+^ extrusion. We measured the initial rate of pH_i_ recovery as a measure of apparent NH_3_:H^+^ efflux primarily attributed to Slc4a11 activity. We observed a rapid recovery in *Slc4a11*^+/+^ MCECs, but significantly slower recovery in *Slc4a11*^−/−^ MCECs (Fig. 4B). To more accurately represent the Slc4a11-mediated NH_3_:H^+^ efflux, we performed further analysis by subtracting the Na^+^/H^+^ exchanger-mediated apparent pH_i_ recovery (Fig. 3C), to obtain the adjusted NH_3_:H^+^ efflux. The average of apparent Na^+^/H^+^ exchanger activity (0.0014/s) between *Slc4a11*^+/+^ and *Slc4a11*^−/−^ MCECs was used given there was no statistical significance between the two cell lines. Figure 4C shows that the adjusted NH_3_:H^+^ efflux is 0.0044 ± 0.0015/s (*n* = 5) in *Slc4a11*^+/+^ MCECs, and significantly smaller 0.0005 ± 0.0003/s (*n* = 5, *P* = 0.0347) in *Slc4a11*^−/−^ MCECs.

### Impaired Glutaminolysis Is Present in *Slc4a11*^−/−^ MCECs as Seen in *Slc4a11*^−/−^ CHED Mouse Model

**Figure 5:**
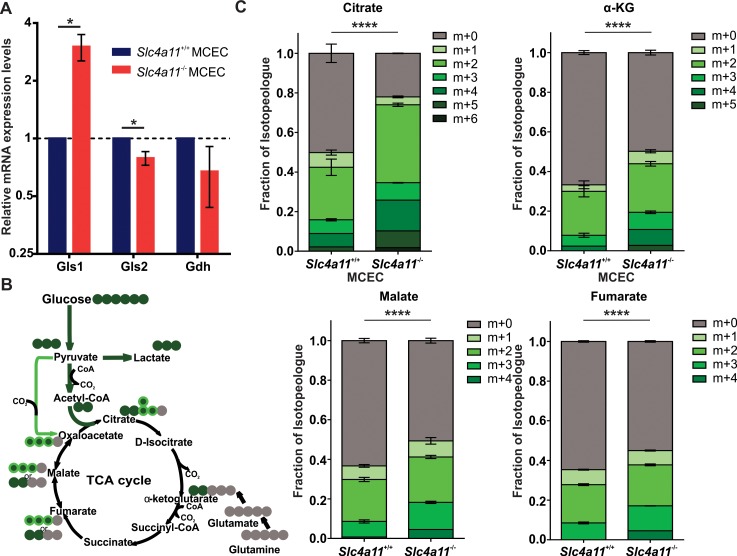
Analysis of glutaminolysis in *Slc4a11*^+/+^ and *Slc4a11*^−/−^ MCECs. (**A**) Real-time quantitative PCR of glutaminolysis enzymes in *Slc4a11*^+/+^ and *Slc4a11*^−/−^ MCECs. There is a 3-fold increase of Gls1 (*P* = 0.013) and a 20% decrease of Gls2 (*P* = 0.031) expression in *Slc4a11*^−/−^ MCECs. Gdh, glutamate dehydrogenase. (**B**) Schematic of the TCA cycle. *Green dots* indicate ^13^C, whereas *gray dots* are ^12^C. *Dark green–colored* ^13^C enters the TCA cycle as acetyl-CoA through pyruvate dehydrogenase, while *light green–colored* ^13^C potentially enters the TCA cycle as oxaloacetate through pyruvate carboxylase, and *gray-colored* ^12^C enters the TCA cycle through glutamine. (**C**) Fraction of ^13^C-labeled TCA cycle intermediates (*green shading*) from cells fed with U-^13^C-glucose in the presence of unlabeled glutamine is significantly higher in *Slc4a11*^−/−^ MCECs than in *Slc4a11*^+/+^ MCECs (33°C, IFN-γ [+]), indicating the glutamine-originated carbon source (*gray*) is significantly reduced. *P < 0.05; ****P < 0.0001.

Many of the enzymes involved in glutamine metabolism exhibit an aberrant expression pattern in *Slc4a11*^−/−^ mouse corneal endothelium,^21^ although the kidney-type glutaminase 1 (Gls1) is upregulated 3-fold in *Slc4a11*^−/−^ mouse corneal endothelium, the liver-type glutaminase 2 (Gls2) can no longer be detected in these cells. Therefore, we set out to determine if the *Slc4a11*^−/−^ MCEC recapitulates this expression difference and manifests as functional differences in glutamine metabolism. Consistent with the observation in mouse corneal endothelial tissue in vivo, we observed an upregulation of Gls1, and a downregulation of Gls2 mRNA expression in *Slc4a11*^−/−^ MCECs (Fig. 5A). These changes in gene expression were also evident at the level of metabolic flux. Our previous studies demonstrated that glutamine supplies approximately 50% of TCA cycle carbon chains in human corneal endothelium.^21^ Consistent with this earlier observation, we find that 50% of citrate and approximately 35% of α-ketoglutarate (α-KG), fumarate, and malate carbon chains were labeled with U-^13^C-glucose-sourced carbon in *Slc4a11*^+/+^ MCECs in the presence of unlabeled glutamine (Fig. 5C). Figure 5B shows the schematic of TCA cycle intermediates analyzed, where ^13^C isotope-labeled green in the setting where cells were cultured with U-^13^C-glucose and unlabeled glutamine. In contrast to wild type, Slc*4a11*^−/−^ MCECs exhibit a significant increase in the percentage of TCA cycle intermediates that contain carbons derived from U-^13^C-glucose, with approximately 75% of citrate and approximately 50% of α-KG, fumarate, and malate carbon chains being labeled with U-^13^C-glucose-sourced carbon. These results indicate that the contribution of glutamine to the TCA cycle is decreased in *Slc4a11*^−/−^ MCECs relative to *Slc4a11*^+/+^ MCECs and support our previous observation that SLC4a11 is essential for facilitating glutaminolysis.

## Discussion

Here we report the generation of the first immortalized MCECs. The use of *H-2Kb*-tsA58 transgenic Immortomouse not only circumvents the limitations and uncertainties associated with in vitro transfection-based immortalization (e.g., initial requirement of large number of cells, different sites of gene integration, multiple copy numbers), but also allows for the production of genetically matched cell lines directly from a transgenic mouse model: *Slc4a11*^+/+^ MCECs and *Slc4a11*^−/−^ MCECs. The conditional immortalization approach provided another advantage that the expression of SV40 large T antigen can be eliminated by simple temperature maneuver and/or removal of IFN-γ. We found that the SV40 large T antigen declined and could be virtually eliminated (Fig. 2B) in the semipermissive condition (IFN-γ [+], 33°C). This condition was preferred because it allowed repeated expansion of MCECs, avoided large cell losses, yet retained endothelial morphology and transport function.

Analysis of ion transporter activity in the two lines of MCECs show that apparent bicarbonate transport, Na^+^/H^+^ exchanger, and lactate transport activities were not significantly changed by *Slc4a11* knockout (Fig. 3). As expected, *Slc4a11*^−/−^ MCECs showed significantly less NH_3_:H^+^ flux relative to *Slc4a11*^+/+^ MCECs (Fig. 4), consistent with the known NH_3_:H^+^ permeability provided by SLC4A11.^27^

The impaired ability to facilitate NH_3_:H^+^ transport in *Slc4a11*^−/−^ MCECs is likely the cause of observed changes in glutamine metabolism. Ammonia was reported to inhibit both the N-ethylmaleimide-sensitive and -insensitive fraction of glutaminase.^39,40^ In *Slc4a11*^−/−^ MCECs, expression changes of glutaminolysis enzymes is consistent with our observation in *Slc4a11*^−/−^ CHED mouse corneal endothelium tissue.^21^ Further analysis of TCA cycle intermediates in *Slc4a11*^+/+^ MCECs indicated that 50% of citrate and approximately 35% of other TCA cycle intermediates were derived from glucose, which is similar to what was found in an immortalized human corneal endothelial cell line.^21^ However, in *Slc4a11*^−/−^ MCECs, we found a significantly increased fraction from glucose-sourced carbon, indicating reduced fraction from glutamine (Fig. 5C), providing evidence that there are functional metabolic changes associated with the enzyme expression changes. Given that ammonia inhibits glutaminase activity,^39^ this observed reduced flux of glutaminolysis suggests that the increased expression of Gls1 found in the *Slc4a11*^−/−^ MCECs (Fig. 5A) may be a compensatory response to glutaminase inhibition.

Interestingly, both human *SLC4A11* and murine *Slc4a11* genes were reported to be a target of p53,^41^ a main cell metabolism regulator.^42^ Conversely, knock-down of *Slc4a11* in bovine nucleus pulposus cells inhibits p53 activity by abolishing p53 phosphorylation.^43^ This suggests that the SLC4A11 transporter could indirectly regulate glutaminolysis enzyme expression via p53 phosphorylation, which is consistent with the finding that glutaminase *GLS2* is a target of p53.^44^

Additionally, we observed fibroblast-like morphology changes only in *Slc4a11*^+/+^ MCECs when tsTAg was induced by IFN-γ (Fig. 1C). Given the direct inhibition from tsTAg on Rb and p53 protein,^45^ it will be of interest to study if the SLC4A11 transporter plays a role in corneal endothelial cell proliferation, polarity formation, and EMT transformation.

In summary, we successfully established two genetically matched conditionally immortal MCECs: *Slc4a11^+/+^* MCECs and *Slc4a11^−/−^* MCECs. These cells remain proliferative with reasonable endothelial morphology after prolonged culture, and present key active ion transport activities as expected from corneal endothelial cells. *Slc4a11*^−/−^ MCECs recapitulate glutaminolysis enzyme expression changes as seen in *Slc4a11*^−/−^ mouse corneal endothelial tissue. These cell lines allowed us to carry out further investigations using cellular-based approaches requiring a significantly larger sample volume than mouse tissue can achieve. Further analysis of TCA cycle intermediates suggests functionally impaired glutaminolysis in *Slc4a11*^−/−^ MCECs. These *Slc4a11*^+/+^ MCEC and *Slc4a11*^−/−^ MCEC cell lines provide an excellent tool for future cell-based experiments to study the pathophysiological changes resulting from loss of Slc4a11 function and for potential therapeutic pharmaceutical reagent screening.
